# Book Review: Awareness Is Freedom: The Adventure of Psychology and Spirituality

**DOI:** 10.3389/fpsyg.2019.02814

**Published:** 2019-12-13

**Authors:** Rémi Thériault

**Affiliations:** Department of Psychology, Université du Québec à Montréal, Montreal, QC, Canada

**Keywords:** psychology, spirituality, self-help, meditation, mindfulness

## Eight Lessons for Self-Growth

In *Awareness is Freedom: The Adventure of Psychology and Spirituality*, Ivtzan (2015) bridges the gap between these two traditions by arguing convincingly that they are not merely compatible, but complementary. The back-cover states that its purpose is “to support readers in their personal journey of self-growth,” placing it squarely in the self-help genre. For Ivtzan, some aspects of our psychological functioning often imprison us, but meditation can help us develop the ability to remain in a state of awareness, which he considers the key to authentic freedom. The book targets a lay audience, as it covers empirical research sparingly and at times seems to rely on the personal views of the author. When Ivtzan does venture into established spiritual discourses, he borrows notions and practices primarily from Buddhism and Yoga, necessarily leaving out other traditions. Unique exercises and psychological tests accompany each of eight lessons.

In lesson one, Ivtzan compares psychology and spirituality and introduces the notion that our “ego”—an illusory sense of personality comprising our beliefs, expectations, and desires—alters our perceptions of reality. As such, Ivtzan adopts a very delimited definition of spirituality: a tool that permits self-growth via the transcendence of the ego. In lesson two, he presents the “royal road” to transcendence, meditation (i.e., non-analytical attentional focusing), its varieties, and benefits. Lesson three introduces us to aware and unaware thought processes, supporting the popular adage that the mind is a good servant but a terrible master. The author makes the case that learning to pay attention without reacting is therefore essential to regaining control over our mind. In lesson four, Ivtzan describes the illusory nature of the ego and proposes instead to embrace our “authentic self.” Lesson five explores how psychological biases color our everyday perceptions in life. According to this view, the human mind organizes incoming information in cognitive “schemas” to help us predict how things should be. In turn, these schemas act as interpretative filters that automatically label situations as either “good” or “bad,” causing us to act in ways that often lead to needless suffering. In lesson six, Ivtzan examines the art of being present and outlines different families of meditation techniques. Lesson seven aims to develop an experiential understanding of impermanence, a fundamental Buddhist concept. Finally, in lesson eight, Ivtzan describes how thoughts, emotions, and body sensations interact in complex ways to define our everyday experience, and how spirituality provides guidance to master our mind and achieve true freedom. Overall, these lessons form an appealing and coherent regimen informed by psychology that guides the reader in a step-by-step process of understanding and changing the mind's habits and patterns in order to live a better and more meaningful life.

## Spirituality Before Science

Despite growing interest, spirituality remains a vast underexplored terrain in psychology. Ivtzan accomplishes the challenging task of making sense out of several fundamental spiritual concepts, and organizing them in accessible lessons. The scope of the book is both a strength and a weakness: the spiritual connoisseur will find the information at times redundant and at other times refreshing; the lay individual will appreciate the book's comprehensiveness but may feel overwhelmed. Prioritizing more specific ideas would facilitate comprehension and increase the effectiveness of this work. For example, the ubiquitous presence of psychological tests and scales comprises an original, but perhaps suboptimal, initiative to integrate more “psychology” along the journey. While these tests do convey a certain form of authority, readers may find their practicality limited and their presence an effective distraction from the core teachings. In my opinion, there would be more effective ways to further bridge the gap between spirituality and psychology. For instance, it would have seemed appropriate to explicitly acknowledge existing thinking in humanistic psychology when discussing the authentic self and self growth (e.g., Medlock, 2012; D'Souza and Gurin, 2016), and transpersonal psychology when describing self-transcendence (e.g., Daniels, 2001; Hartelius et al., 2007). Likewise, including classical Self-Determination Theory studies and theorization demonstrating the basic psychological need for autonomy (Ryan and Deci, 2000, 2006) would have helped support the premise that we fundamentally strive for “freedom.” Finally, it would also have seemed fit to more strongly link spiritual traditions emphasizing the power of the mind and of intention by briefly covering some of the social-psychological literature on the topic; for instance, to describe and integrate the concepts of expectation and placebo effects (e.g., Madon et al., 2011; Crum and Phillips, 2015), mindsets (Dweck, 2006; Burnette et al., 2013), and implementation intentions (Gollwitzer, 1999; Gollwitzer and Sheeran, 2006), to name just a few.

Other exercises presented in the book, such as mindfulness, may be more promising for inducing positive change (Eberth and Sedlmeier, 2012; Ivtzan and Lomas, 2016; but see Goyal et al., 2014). Unfortunately, some of the proposed exercises are currently not empirically supported. Take, for instance, the inclusion of meditation techniques involving “chakras,” hypothetical energy centers located across the body. While we cannot dismiss the potential therapeutic benefits associated with such practices, the promotion of unproven therapies in the name of psychology does raise some concerns regarding our responsibility as scientists and practitioners. Besides, the book references 18 experimental studies and two meta-analyses for the full eight lessons (equivalent to under 3 references per section on average)—a humble count considering its claim of being “backed up by scientific findings that enhance the legitimacy and power of its message” (back cover). Therefore, readers may remain unclear as to what, exactly, rests on evidence and what emerges from personal experience or spiritual beliefs.

## Conclusion: A First Step on the Path

In summary, Itai Ivtzan wears two hats: he researches positive psychology at Naropa University/the University of East London during the week and runs meditation retreats on the weekends. Yet, the skills of the spiritual master stand out more prominently than those of the psychological researcher. Despite its limitations, this practical handbook could conceivably complement personal development-oriented psychotherapies or relevant undergraduate courses, given the book's drawbacks are clearly explored and discussed—especially if new evidence arises to support the proposed exercises and theoretical frameworks.

## Author Contributions

The author confirms being the sole contributor of this work and has approved it for publication.

### Conflict of Interest

The author declares that the research was conducted in the absence of any commercial or financial relationships that could be construed as a potential conflict of interest.

## References

[B1] Burnette J. L. O'Boyle E. H. VanEpps E. M. Pollack J. M. Finkel E. J. (2013). Mind-sets matter: a meta-analytic review of implicit theories and self-regulation. Psychol. Bull. 139, 655–701. 10.1037/a0029531 22866678

[B2] Crum A. Phillips D. J. (2015). Self-fulfilling prophesies, placebo effects, and the social-psychological creation of reality, in Emerging Trends in the Social and Behavioral Sciences, eds Scott R. A. Kosslyn S. M. (Hoboken, NJ: John Wiley and Sons), 1–14. 10.1002/9781118900772.etrds0296

[B3] Daniels M. (2001). On transcendence in transpersonal psychology. Transpers. Psychol. Rev. 5, 3–11.

[B4] D'Souza J. Gurin M. (2016). The universal significance of Maslow's concept of self-actualization. Human. Psychol. 44, 210–214. 10.1037/hum0000027

[B5] Dweck C. S. (2006). Mindset: The New Psychology of Success. New York, NY: Random House.

[B6] Eberth J. Sedlmeier P. (2012). The effects of mindfulness meditation: a meta-analysis. Mindfulness 3, 174–189. 10.1007/s12671-012-0101-x

[B7] Gollwitzer P. M. (1999). Implementation intentions: strong effects of simple plans. Am. Psychol. 54, 493–503. 10.1037/0003-066X.54.7.493

[B8] Gollwitzer P. M. Sheeran P. (2006). Implementation intentions and goal achievement: a meta-analysis of effects and processes. Adv. Exp. Soc. Psychol. 38, 69–119. 10.1016/S0065-2601(06)38002-1

[B9] Goyal M. Singh S. Sibinga E. M. S. Gould N. F. Rowland-Seymour A. Sharma R.. (2014). Meditation programs for psychological stress and well-being: a systematic review and meta-analysis. JAMA Intern. Med. 174, 357–368. 10.1001/jamainternmed.2013.13018 24395196 PMC4142584

[B10] Hartelius G. Caplan M. Rardin M. A. (2007). Transpersonal psychology: defining the past, divining the future. Human. Psychol. 35, 135–160. 10.1080/08873260701274017

[B11] Ivtzan I. (2015). Awareness is Freedom: The Adventure of Psychology and Spirituality. Winchester: Changemakers Books.

[B12] Ivtzan I. Lomas T. (2016). Mindfulness in Positive Psychology: The Science of Meditation and Wellbeing. London: Routledge 10.4324/9781315747217

[B13] Madon S. Willard J. Guyll M. Scherr K. C. (2011). Self-fulfilling prophecies: mechanisms, power, and links to social problems. Soc. Personal. Psychol. Compass 5, 578–590. 10.1111/j.1751-9004.2011.00375.x

[B14] Medlock G. (2012). The evolving ethic of authenticity: from humanistic to positive psychology. Human. Psychol. 40, 38–57. 10.1080/08873267.2012.643687

[B15] Ryan R. M. Deci E. L. (2000). Self-determination theory and the facilitation of intrinsic motivation, social development, and well-being. Am. Psychol. 55, 68–78. 10.1037/0003-066X.55.1.68 11392867

[B16] Ryan R. M. Deci E. L. (2006). Self-regulation and the problem of human autonomy: does psychology need choice, self-determination, and will? J. Personal. 74, 1557–1586. 10.1111/j.1467-6494.2006.00420.x 17083658

